# Albuterol-Induced Type B Lactic Acidosis: Not an Uncommon Finding

**DOI:** 10.7759/cureus.8269

**Published:** 2020-05-25

**Authors:** Sreenath Meegada, Vijayadershan Muppidi, Suman Siddamreddy, Tejo Challa, Shravan K Katta

**Affiliations:** 1 Internal Medicine, University of Texas Health Science Center/Christus Good Shepherd Medical Center, Longview, USA; 2 Internal Medicine, Indiana University Health, Indianapolis, USA; 3 Internal Medicine, Baptist Health Medical Center, Little Rock, USA; 4 Internal Medicine, Texas Health Arlington Memorial Hospital, Arlington, USA; 5 Internal Medicine, University of Texas, Arlington, USA

**Keywords:** albuterol, type b lactic acidosis, lactic acidosis

## Abstract

Lactic acidosis (LA) is usually a medical emergency diagnosed by laboratory evaluation in emergency rooms (ERs) and hospital settings in critically ill patients. LA is classified into two major types based on pathophysiology; type A results from tissue hypoxia and/or hypoperfusion and type B results from deranged metabolic activity in the cells in the absence of hypoxia/hypoperfusion. Prompt evaluation and treatment are essential to prevent morbidity and mortality, especially in patients with type A LA. Most cases of LA are due to type A (hypoperfusion/hypoxia). However, with increased testing of lactic acid levels in ERs and hospitals, we are encountering a few cases of type B LA as well. Diagnosing the exact type is crucial because of differences in management. We here describe a patient with albuterol-induced type B LA, which resolved after discontinuing the albuterol breathing treatments.

## Introduction

Lactic acidosis (LA) is defined as elevated lactate levels greater than 4 millimoles/liter, and hyperlactatemia if greater than 2 millimoles/liter [1,2]. The elevated lactic acid level is either by increased production or by decreased utilization/clearance from the body or deranged cellular metabolism. The incidence of LA has recently surged with new sepsis protocol/guidelines followed in emergency rooms and hospitals on critically ill patients. Based on pathophysiology, LA is classified into two types; Type A LA from hypoperfusion/hypoxia and type B LA from deranged metabolism within cells [3]. On the contrary, the incidence of elevated lactic acid levels has been up trending in patients who are not septic or in shock, which is type B LA.

## Case presentation

A 63-year-old Caucasian woman with a medical history of asthma, chronic obstructive pulmonary disease (COPD), diabetes mellitus type 2, hypertension presented to the emergency room (ER) with shortness of breath and cough. She started having shortness of breath for 48 hours present at rest, worse with exertion. She denied orthopnea, paroxysmal nocturnal dyspnea, swelling of feet or body, and chest pain. Cough is productive with greenish sputum. She normally did not have a productive cough. She denied any sick contacts, fevers, chills, or rigors. She quit smoking 30 years ago but had 25 years of one pack per day smoking. She is not an alcoholic and never tried illicit drugs. Her home medications include albuterol rescue inhaler as needed, budesonide/formoterol inhaler, glipizide, aspirin, hydrochlorothiazide, benzonatate as needed, singular, and losartan.

On initial evaluation in ER, the patient was afebrile, tachypneic at 32 per minute, blood pressure of 177/113 mmHg, oxygen saturation of 98% on room air. On examination, the patient was in respiratory distress, getting nebulized treatments. She was alert, oriented, and in acute distress, as mentioned. Lung exam was significant for decreased breath sounds with end-expiratory wheezes bilaterally; the patient was using accessory muscles. Heart sounds were normal, no murmur. The abdomen was benign, soft, non-tender. The neurological exam was normal. The rest of the exam was normal.

**Figure 1:**
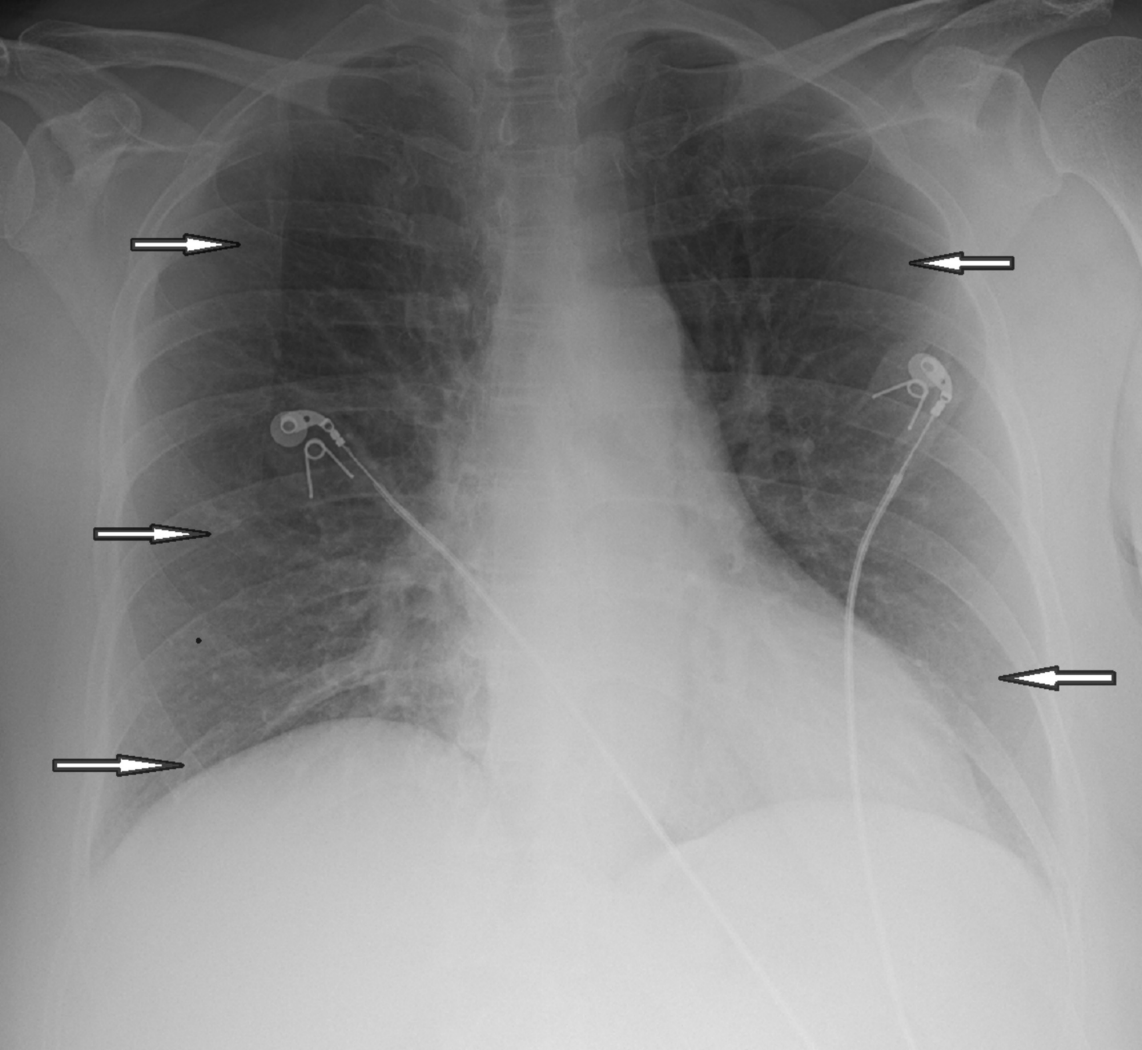
Chest X-ray showing emphysematous changes, no focal consolidation (arrows pointing)

**Figure 2:**
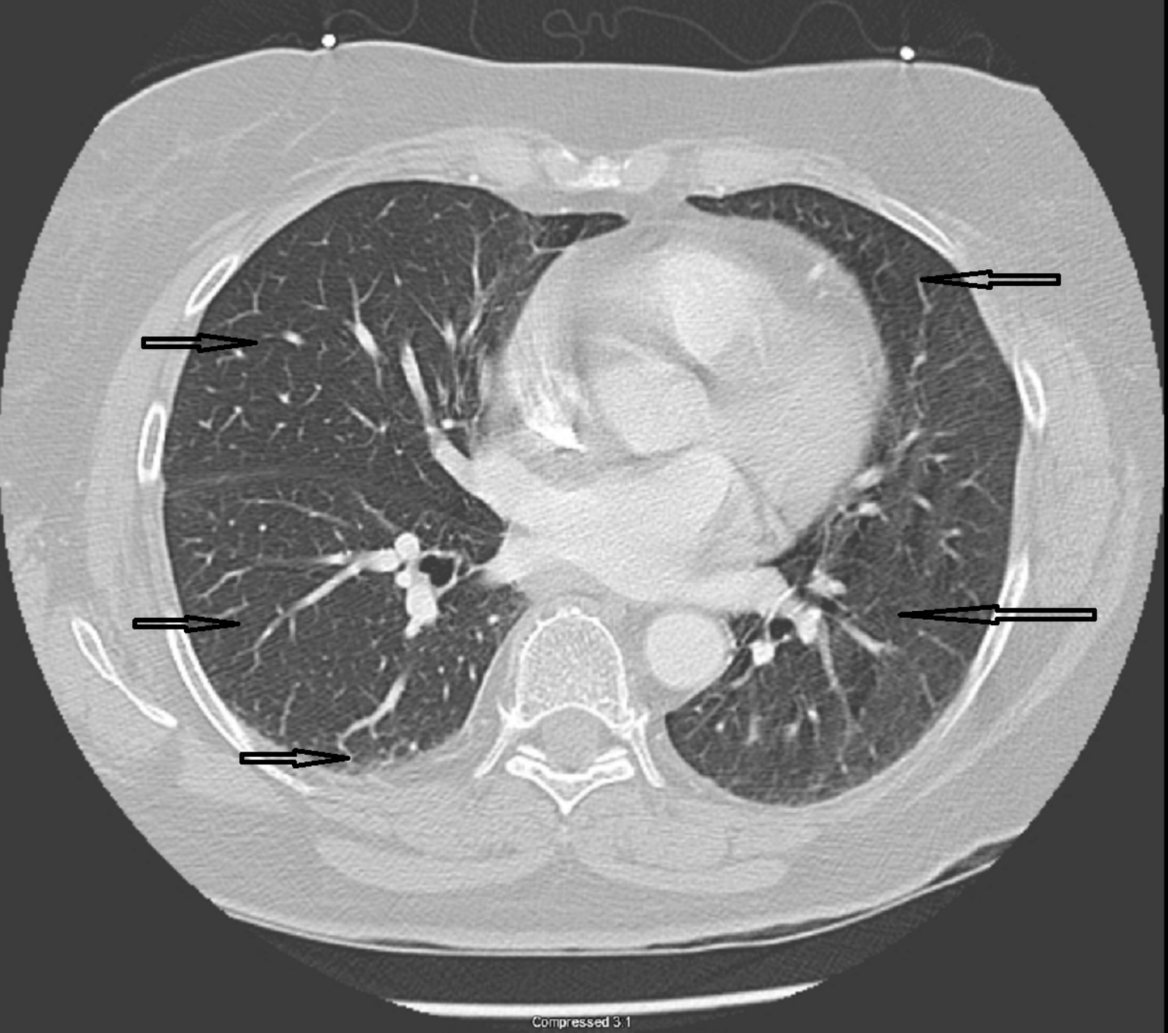
Computed tomography scan of chest showed emphysematous changes with no pulmonary embolism or aortic dissection (arrows pointing)

Lab evaluation showed normal complete blood cell count, and the metabolic panel was significant for hypokalemia. The initial lactic acid level was 2.2, which went up to 4.7 in four hours. Chest X-ray showed emphysematous changes, no focal consolidation after which computed tomography of lungs was done with intravenous contrast, which showed emphysematous lung parenchyma, no pneumonia, no pulmonary emboli, and no pneumothorax (Figures 1-2).

Due to elevated lactic acid levels, the patient was started on sepsis protocol from ER. The patient received broad-spectrum antibiotics, intravenous fluid resuscitation, steroids, bronchodilators (albuterol/ipratropium), and admitted to hospitalist service.

During his course in the hospital, the patient’s symptoms significantly improved; however, his lactate levels were trending up 2.2-->4.7-->6.7-->4.4-->3.9-->4.1-->6.1-->5.1 millimoles/liter. At this point, we had to re-evaluate the causality for persistently elevate lactic acid levels. Considering the patient’s clinical improvement, we thought it could be type B LA induced by albuterol. We held albuterol nebulizing treatments and continued only ipratropium after which lactic acid levels down trended and became normal in few hours as follows: 4.7-->2.4-->2.0-->1.7 millimoles/liter (Table 1). 

**Table 1:** Trend of lactic acid levels (please note downtrend after stopping albuterol)

Date and time	Lactic acid levels in Millimoles/Liter
2/14/19, 7 PM	2.2
2/14/19, 9 PM	4.7
2/14/19, 11:15 PM	6.7
2/15/19, 1 AM	4.4
2/15/19, 3 AM	3.9
2/15/19, 5 AM	6.1
2/15/19, 7 AM (Albuterol stopped)	5.1
2/15/19, 10 AM	4.7
2/15/19, 1230 PM	2.4
2/15/19,4 PM	2.0
2/15/19,8 PM	1.7

The patient was discharged home in a stable condition with a diagnosis of COPD exacerbation and type B LA induced by albuterol.

## Discussion

Classification

LA is mainly classified based on the pathophysiology of generating excess lactic acid levels in the body. It is broadly divided into two types: type A, conditions associated with impaired tissue oxygenation, and type B, conditions in which tissue oxygenation is not apparent. Most of the time, these two conditions co-exist [4]. There is a rare entity called D-LA, which is commonly seen in short gut syndrome or gut malabsorption syndromes [5].

Causes and pathophysiology of LA

Type A LA is caused by hypoxia, hypoperfusion, shock, cardiopulmonary arrest and type B LA is caused by drugs, liver failure, kidney failure, diabetes type 2, malignancy, seizures, catecholamines, beta-agonists, thiamine deficiency and so on (Table 2) [6].

**Table 2:** Causes of lactic acidosis [6]

Type A lactic acidosis	Hypoxia, Hypoperfusion, Shock, Cardiopulmonary arrest
Type B lactic acidosis	Metformin Alcoholic and diabetic ketoacidosis, Liver failure, Kidney failure, Malignancy, Seizures, Catecholamine, Beta-agonists, Thiamine deficiency
D-lactic acidosis	Small gut syndrome, Gut malabsorption

Normal healthy individual generates up to 20 millimoles/kg of lactic acid in 24 hours from the glycolytic pathway. It is primarily (80%) converted into carbon dioxide and water, and up to 20% to generate glucose. Lactic acid is mainly utilized in the liver, less extent in other organs. Pyruvate converting into lactate by enzyme lactate dehydrogenase is the key chemical reaction in generation of lactic acid [7]. Lactic acid is accumulated in the body by several mechanisms, major ones being: increased pyruvate production, decreased cellular cytoplasmic redox state which favors pyruvate to lactate conversion, and decreased entry of pyruvate into mitochondria [8,9]. 

Medication-induced type B LA is more diagnosed in hospital settings because of introduction of sepsis protocols. Some common causes of type B LA are metformin, alcoholism, cancer, antibiotics like linezolid, anti-depressants, and beta-agonists [10]. 

Type B LA in patients with asthma and COPD exacerbation

Scheduled beta-agonists (albuterol) combined with anticholinergic agents (ipratropium) is the standard therapy for patients admitted with asthma or COPD exacerbation [11]. Type B LA has been reported in patients treated with inhaled or nebulized beta-agonists admitted for bronchospasm from asthma or COPD exacerbation. The mechanism of LA in these patients has not been clearly understood and could be multifactorial. Beta-agonists induce glycolysis and lipolysis, which increases the production of pyruvate and free fatty acids. Pyruvate converts into lactate and free fatty acids inhibit pyruvate dehydrogenase and inhibit mitochondrial pyruvate uptake [12]. Fatigued respiratory muscles also increase lactic acid levels, but there is only indirect evidence to this hypothesis [13]. Intra-venous beta-agonists like terbutaline are known to cause elevated lactate levels, which validates the role of inhaler beta-agonists in type B LA [14].

Our patient had albuterol-induced type B LA after ruling out all other causes, he was hemodynamically stable, and his LA normalized after stopping albuterol therapy.

Treatment

Treat the source of LA, which includes restoring perfusion and oxygenation in type A LA and stopping the offending agent in type B LA. Short term administration of intravenous bicarbonate can be helpful in treating critical metabolic acidosis induced by LA. 

## Conclusions

Type B LA is most often diagnosed because of new algorithms set up by ERs and hospitals as a part of the sepsis protocol. Type B LA should be considered in the absence of tissue hypoperfusion and hypoxia. Albuterol-induced LA is likely from activated glycolysis and lipolysis which in turn increases pyruvate and free fatty acids leading to increased lactic acid production.
